# Effects of Alprazolam on Cortical Activity and Tremors in Patients with Essential Tremor

**DOI:** 10.1371/journal.pone.0093159

**Published:** 2014-03-25

**Authors:** Jaime Ibáñez, Jesús González de la Aleja, Juan A. Gallego, Juan P. Romero, Rosana A. Saíz-Díaz, Julián Benito-León, Eduardo Rocon

**Affiliations:** 1 Bioengineering Group, Consejo Superior de Investigaciones Científicas, Arganda del Rey, Spain; 2 Department of Neurology, University Hospital “12 de Octubre”, Madrid, Spain; 3 Department of Medicine, Faculty of Medicine, Complutense University, Madrid, Spain; 4 Centro de Investigación Biomédica en Red sobre Enfermedades Neurodegenerativas, Madrid, Spain; 5 Universidad Francisco de Vitoria, Pozuelo de Alarcón, Spain; Toronto Western Hospital, Canada

## Abstract

**Background:**

Essential tremor (ET) is characterised by postural and action tremors with a frequency of 4–12 Hz. Previous studies suggest that the tremor activity originates in the cerebello-thalamocortical pathways. Alprazolam is a short-acting benzodiazepine that attenuates tremors in ET. The mechanisms that mediate the therapeutic action of alprazolam are unknown; however, in healthy subjects, benzodiazepines increase cortical beta activity. In this study, we investigated the effect of alprazolam both on beta and tremor-related cortical activity and on alterations in tremor presentation in ET patients. Therefore, we characterised the dynamics of tremor and cortical activity in ET patients after alprazolam intake.

**Methods:**

We recorded hand tremors and contralateral cortical activity in four recordings before and after a single dose of alprazolam. We then computed the changes in tremors, cortico-muscular coherence, and cortical activity at the tremor frequency and in the beta band.

**Results:**

Alprazolam significantly attenuated tremors (EMG: 76.2±22.68%), decreased cortical activity in the tremor frequency range and increased cortical beta activity in all patients (*P*<0.05). At the same time, the cortico-muscular coherence at the tremor frequency became non-significant (*P*<0.05). We also found a significant correlation (*r* = 0.757, *P*<0.001) between the reduction in tremor severity and the increased ratio of cortical activity in the beta band to the activity observed in the tremor frequency range.

**Conclusions:**

This study provides the first quantitative analysis of tremor reduction following alprazolam intake. We observed that the tremor severity decreased in association with an increased ratio of beta to tremor-related cortical activity. We hypothesise that the increase in cortical beta activity may act as a blocking mechanism and may dampen the pathological oscillatory activity, which in turn attenuates the observed tremor.

## Introduction

Essential tremor (ET) is a movement disorder caused by a central oscillatory network and is characterised by postural and action tremors ranging in frequency from 4–12 Hz [1]. The exact mechanisms of tremor generation in essential tremor (ET) are still unknown [2], [3]. However, a number of studies implicate a neuronal loop involving cerebello-thalamocortical pathways as the locus of tremorogenesis [4]–[9]. In particular, the analysis of coherence between cortical and muscle activity has demonstrated the presence of tremor-related activity in cortical structures [6] and has provided a method for studying the change in cortico-muscular interaction over time [7].

Pharmacological treatments for ET remain limited and are only partly effective [1], [10]. The action mechanisms of these drugs are unspecified, although it is assumed that they attenuate tremors by interfering with the widespread pathological oscillations that occur throughout the motor system [11]. Alprazolam, which is one of several pharmacological alternatives for the treatment of ET, is a short-acting benzodiazepine with an accepted classification of probably efficacious (level B) agent [12]–[14].

Previous studies with healthy subjects have reported increased cortical beta rhythm activity after the intake of other benzodiazepines [15]–[18]. It is known that benzodiazepines increase the affinity of the γ-aminobutyric acid (GABA)-A receptor for its neurotransmitter, which increases the size of the inhibitory postsynaptic potentials that are generated by GABA neurotransmission [19]. However, it is unclear how enhancing inhibition may increase the power of the beta and gamma rhythms [18], [20].

Because voluntary motor commands are projected to the targeted motor unit populations in the beta band [21]–[23], we hypothesised that the increase in cortical beta activity due to benzodiazepines would alter the transmission of descending motor commands. Furthermore, we expected that such an increase in oscillatory beta activity would impede the appearance of pathological tremor-related cortical activity. Therefore, we analysed the interplay between cortical activity in the beta band and tremor frequency after alprazolam intake to discern how this interaction was associated with the drug effects on tremor and cortico-muscular coupling at the tremor frequency.

## Methods

### Patients

Eight patients (two females, age 64.1±13.2 years; mean ± SD) were included from a general neurology outpatient clinic (the details are provided in Table 1). All patients were diagnosed with ET according to the Movement Disorders Society Diagnostic Criteria [24]. We excluded patients with severe tremors of the hands or the head to avoid interference with the recordings. None of the patients had other neurological conditions apart from ET or suffered from psychiatric disorders. Additionally, none of the patients were taking medication to treat their tremors or any other drugs that could alter their tremors. The experimental protocol was approved by the Ethical Committee of the University Hospital “12 de Octubre” (Madrid) and was in accordance with the Declaration of Helsinki. All participants provided written informed consent.

**Table 1:** Main baseline demographic and clinical variables.

Patient	01	02	03	04	05	06	07	08	09
Gender	Male	Female	Male	Female	Male	Male	Male	Male	Male
**Age (years)**	76	80	44	63	45	65	77	69	58
**ET family history**	Y	Y	Y	Y	Y	Y	Y	N	N
**Disease duration (y)**	5	32	15	7	4	10	2	3	4
**Dominant side of tremor**	L	R	R	L	R	L	L	L	R
**EMG tremor freq. (Hz)**	6.2	5.2	7.0	6.2	–	6.2	7.0	6.2	8.2
**Leg tremor**	N	Y	N	Y	N	N	N	N	N
**Head tremor**	N	N	N	Y	Y	Y	N	N	N
**ETRS**	45	32	17	38	18	15	14	16	22

Fahn, Tolosa, Marin Essential Tremor Rating Scale (ETRS).

### Recordings

Wrist tremors on the most affected side were measured with solid-state gyroscopes and surface EMG. Two gyroscopes (Technaid S.L., Madrid, Spain), placed on the hand dorsum and the distal third of the forearm, measured wrist tremors by computing the difference between them [25], [26]. The data were sampled at 50 Hz.

Surface EMG was recorded using a grid of 13×5 electrodes (1 missing electrode), with 8 mm inter-electrode distance (LISiN–OT Bioelettronica, Torino, Italy). The electrode grid was placed on the wrist extensors and centred on the muscle exhibiting the clearest tremorogenic activity; the common reference was set to the wrist using a humidified bracelet. The data were amplified (EMGUSB, OT Bioelettronica, Torino, Italy), band-pass filtered (10–750 Hz), and sampled at 2,048 Hz.

EEG signals were recorded from 16 positions (F2, F4, FCz, FC2, FC4, FC6, Cz, C2, C4, C6, T8, CP2, CP4, CP6, Pz and P4, according to the International 10–20 system, when the left arm was recorded; the symmetric positions were employed when the right arm was recorded) using passive Au electrodes. We recorded the cortical activity from the contralateral hemisphere because the cortico-muscular coherence at the tremor frequency [6], [7], [27], [28] and within the beta band [23], [29] are best observed at this location. The reference was set to the common voltage of the two earlobes. AFz was used as ground. The signal was amplified (gUSBamp, g.Tecgmbh, Graz, Austria), band-pass (0.5–60 Hz) and notch filtered (50 Hz), and sampled at 256 Hz.

The recording systems were synchronised with a common digital signal. The data were analysed offline using Matlab (The Mathworks Inc., Natick MA, USA) and SPSS (IBM, Chicago, Illinois).

### Procedure

The study was performed in a sound and light-attenuated room. Patients sat in a comfortable chair with their arms supported. During the measurement phase, all patients were instructed to remain relaxed with their eyes open and their gaze fixated on a point on the wall. Patients were instructed not to eat or drink anything (water was allowed) for 2 h before the recordings. To evaluate the effects of alprazolam, patients underwent four, 4–min recording sessions at different time points relative to alprazolam administration as follows: before the administration of alprazolam (Run0), immediately after (Run1), 40 min after (Run2), and 80 min after (Run3). Postural tremors were elicited by asking patients to hold the measured hand outstretched with the palm down and parallel to the ground. In patients who exhibited a very mild tremor before the experiment (patients 02 and 04), weight loads of 0.5 Kg were attached to the hand to enhance the tremor [6], [7].

A single dose of 0.50 mg of alprazolam was administered to patients who weighed less than 75 kg; the remaining five patients received a single dose of 0.75 mg. No patient reported adverse effects. Two patients were excluded due to technical problems with EEG acquisition (patient 03) and to the absence of tremors during the measurement session (patient 05).

### Data Processing and Analysis

The EEG signals were spatially filtered using the Hjorth transform [30]. The resultant channels (FC2, FC4, C2, C4, C6, CP2 and CP4) were used in the subsequent analyses. Artefacts were removed based on visual inspection.

**Figure 1:**
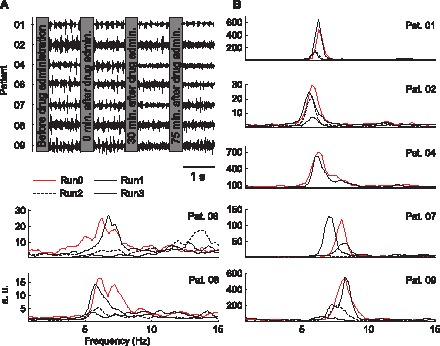
Time course of the tremor power measured with EMG. (A) Time course of the raw EMG (the amplitude scale is the same for all subjects); (B) PSD of the EMG signals (one panel per patient).

After the examination of the amplitude spectra of the gyroscope and EMG data, we defined the tremor frequency range for our group of patients as 4–9 Hz (see Fig. 1). This range was used to estimate both the tremor power (measured with gyroscopes and EMG) and the power of the tremor-related cortical activity.

To select the surface EMG channel that best characterised the tremor, we used the criterion of maximising the signal-to-noise ratio (SNR) of the tremor component of the EMG signal. The SNR was defined as the ratio of the integral of the power spectral density (PSD) within the tremor frequency range to the integral of the PSD of the rest of the signal, similar to [6]. This channel was used throughout the whole analysis.

The percentage of tremor reduction between the first and last runs (Run0 and Run3) was computed by analysing the gyroscope data. Tremor severity was defined as the integral of the PSD of the signal in the tremor frequency range. Before the data were band-pass filtered (2–15 Hz) to extract the tremor [26]. We also calculated how the neural drive to the muscles related to tremors was reduced after alprazolam intake by computing the percentage of the tremor power decrease in Run1, Run2 and Run3 with respect to Run0 for the EMG data.

Cortico-muscular coherence was computed to assess how the tremor-related cortical drive to the muscle varied due to the effect of alprazolam. We calculated the coherence between all the processed EEG channels and the rectified EMG at the electrode previously selected [31], and we chose the EEG channel that exhibited the largest peak at the tremor frequency for the subsequent calculations. We used the method for coherence estimation proposed in [32]: the signals were divided into epochs of 1 s, and their individual spectra and cross-spectra were computed. Coherence was estimated as the ratio between the squared cross-spectrum and the product of the two individual power spectra [6], [32]. The confidence limit was obtained as in [33].

To study how alprazolam affected the tremor-related cortical activity and the cortical activity in the beta band, we assessed the changes in the EEG spectra by calculating the integral of the PSD at the selected channel in the tremor frequency range (4–9 Hz, see above) and in the beta band (13–30 Hz).

### Statistical Analysis

We used the Wilcoxon rank sum test to compare the tremor severity measured by the gyroscopes before (Run0) and 80 min after the administration of alprazolam (Run3).

The Kruskal–Wallis test was used to compare the tremor-related neural drive to the muscle in Run3, Run2 and Run1 with respect to Run0. Significant differences between pairs of data were assessed with the Games-Howell test, assuming non-equal variances. The same test was used to compare the changes in the power of cortical activity in the beta band and in the tremor-frequency range and to compare the change in the ratio between the activities in these two bands. In all cases, changes in Run3, Run2 and Run1 were obtained with respect to Run0.

Finally, we calculated the Spearman’s rank correlation to investigate the relationship between the decrease in tremor severity (in terms of neural drive to the muscle, i.e., EMG) and the change in the ratio between the EEG activity in the beta band and in the tremor frequency range, using the data from Run3, Run2 and Run1 with respect to Run0.

Results are reported as the mean ± SD, and considered significant if *P*<0.05.

## Results

The tremor amplitude as measured with gyroscopes showed a significant (*P* = 0.029) reduction 80 min after the administration of alprazolam (mean: 74.3±30.2%). The mean tremor amplitudes during Run0 and Run3 were 0.31±0.34 rad^2^s^−2^ and 0.043±0.049 rad^2^s^−2^, respectively.

Fig. 1 illustrates the change in tremorogenic muscle activity across the different runs. There was a significant difference in the tremor power reductions observed in Run3, Run2 and Run1 compared to Run0 (*P* = 0.002). Post hoc analysis showed that the decreases in tremor power observed in Run3 (mean: 75.0±17.6%) and in Run2 (mean: 69.3±17.9%) were not significantly different from each other (*P* = 0.82); however, both were significantly larger than that observed in Run1 (mean: 4.6±23.6%) with respect to Run0 (*P*<0.001 and *P*<0.001, respectively).

There were no significant differences (*P* = 0.917, Wilcoxon rank sum test) between the tremor frequency measured with the gyroscopes (6.22±0.53 Hz) and EMG (6.20±0.56 Hz). The tremor frequency did not change during the recordings, although no statistics were obtained because the tremor was not clearly identifiable in some patients after alprazolam intake (see Fig. 1C).

**Figure 2:**
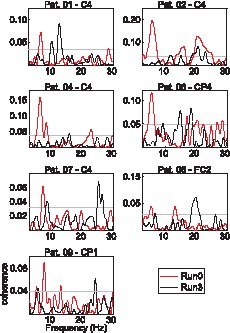
Cortico-muscular coherence. Obtained from Run0 and Run3 (Run2 is included for patient 07 only). Each panel represents a different patient. The significance level (*P*<0.05) is also displayed (solid grey line).

Fig. 2 depicts the coherence between the EEG channel with the largest coherence at the tremor frequency and the selected EMG channel. The coherence at the tremor frequency in Run0 was significant (*P*<0.05) for all patients. In Run3, the coherence at the tremor frequency decreased in all cases and fell below the significance threshold in five out of seven patients. In the case of patient 07, the significant coherence in Run3 was accompanied by the observed rebound of EMG tremor power in Run3 with respect to Run2 (Fig. 1).

**Figure 3:**
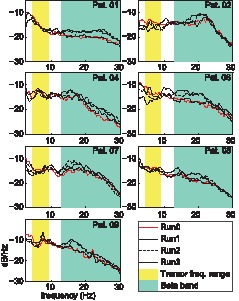
PSDs of the EEG data in the optimal channel for each patient. The shaded areas represent the tremor frequency range (yellow) and the beta band (green), as used to evaluate changes in the cortical activity. Each panel represents a different patient.

Fig. 3 shows the time course of the EEG power spectrum along the runs. Relative to Run0, there was a significant increase in the ratio between the beta and tremor-related cortical activities for Run3, Run2 and Run1 (*P* = 0.003). Post hoc analysis showed no significant difference between Run3 (mean 129.2±96.7%) and Run2 (mean 94.0±48.5%) (*P* = 0.68), but both were significantly larger than Run1 (mean 9.81±17.6%) (*P* = 0.039 and *P* = 0.007, respectively).

**Figure 4:**
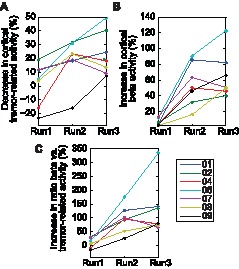
Changes (in %) of the cortical beta and tremor related activities in Run1, Run2, and Run3 with respect to Run0. (A) Decrease in the power of cortical activity within the tremor frequency range. (B) Increase in the power of the beta band. (C) Increase in the ratio between the power in the beta band and in the tremor frequency range.

When considered separately, the observed pattern of changes in cortical beta activity and in cortical activity within the tremor frequency range were similar. The increases in beta power in Run1, Run2 and Run3 with respect to Run0 (5.7±3.6%, 54.4±26.8% and 64.8±29.2% respectively) were significantly different (P = 0.002). Post hoc analyses showed that statistical significance was achieved for the comparison between Run1 and Run2 (*P* = 0.007) and between Run1 and Run3 (*P* = 0.004), whereas the difference between Run2 and Run3 was not statistically significant (*P* = 0.77). The decrease of cortical activity in the tremor frequency range was also significantly different in Run1 (1.7±15.4%), Run2 (18.1±16.2%) and Run3 (23.1±16.1%) with respect to Run0 (*P* = 0.035). However, post hoc analyses of pairwise group comparisons did not achieve statistical significance. A graph of these results is depicted in Fig. 4, where it is shown that, after alprazolam intake, the cortical activity within the tremor frequency range decreases and the cortical beta activity increases. This effect can be observed for all patients 80 min after alprazolam intake.

**Figure 5:**
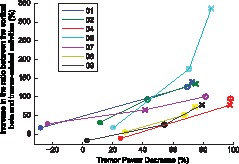
Relationship between changes in tremor power and the ratio between the beta and the tremor-related cortical activities for all patients and runs. Run1, Run2 and Run3 (all with respect to Run0) are represented with the symbols •, ○ and X, respectively.

There was a significant correlation (*r* = 0.757, *P*<0.001) between the tremor power decrease (as measured with EMG) and the increased ratio of beta to tremor-related activity in cortical areas contralateral to the measured hand (in the EEG channel where significant cortico-muscular coupling was best observed, Fig. 4). This relation also held when analysing separately the results of each patient, *i.e.,* all of the patients presented a positive relationship between both variables, as shown in Fig. 5.

## Discussion

This study characterises the dynamics of tremor and cortical activity in ET patients after alprazolam intake. We observed significant changes in the measured tremor as well as in the cortical activity both in the beta band and in the tremor frequency range due to the effects of the drug. These changes revealed a significant correlation between tremor reduction and the relative change in cortical activity in the beta and tremor-related bands.

The study also provides the first quantitative evidence of tremor changes after a single dose of alprazolam. Two previous studies showed a significant improvement in tremor rating scales after treating ET patients with alprazolam. Specifically, these studies reported a 30% reduction in a double-blind placebo-controlled trial [14], a 25% decrease in tremor intensity rated by functional scores and a 46% increase in the global improvement scale by patient self-evaluation [13]. Here, we confirm these results quantitatively by showing a significant tremor reduction of 69.4% and 75.8% according to the EMG data (*i.e.,* the neural input to the muscle related to tremor) acquired 40 and 80 min after a single dose of alprazolam. The timing of the observed effects is also consistent with the time of peak concentration of alprazolam reported for healthy elderly subjects (48±18 min) [34]. The use of a low, single dose of alprazolam was aimed to avoid drowsiness that induced EEG changes consistent with sedation and were not related to the hypothesised anti-tremorogenic effect. In fact, the observed increase of EEG activity in the beta band argues against a sedative effect of alprazolam because beta activity is considered an index of cortical arousal [35].

We assessed the change in strength of cortico-muscular coupling due to the effect of alprazolam. As expected [6], [7], [27], [36], the coherence values at the tremor frequency were significant in all patients before drug intake. The coherence decreased 80 min after alprazolam intake and was below the significance threshold in all patients, except for patients 07 and 08. Interestingly, patient 07 also presented a rebound of tremor severity in Run3 compared to Run2 (according to the EMG data) and demonstrated the highest levels of the cortical beta activity 40 min after drug administration. These data suggest an earlier onset and termination of the effects of alprazolam in this patient. Additionally, we observed a decrease in the power of cortical activity in the tremor frequency range in the EEG channel showing the largest coherence at the tremor frequency. Taken together, these results suggest that the pathological oscillatory activity in the cortex decreased when the drug started to take effect. On the other hand, coherent activity in the beta band was not evident in our results either from the beginning of the session or during subsequent runs. This finding is in contrast with the robust coherence at the beta band observed in other studies; however, here, the patients performed mild contractions to hold their hands extended, which explains the lack of meaningful results in this regard [37], [38].

We observed a significant increase in cortical beta activity in all ET patients during a period after alprazolam intake, consistent with previous research addressing the effects of other benzodiazepines in healthy subjects [16], [17]. Interestingly, the power of the EEG activity at the beta band is also enhanced in alcoholics [39] or after a small single dose of alcohol [40], and in 50–90% of ET patients, alcohol acts to reduce the tremor amplitude [12], [41]. While the precise mode of action of ethanol in ET has not been established [42], its principal effect is likely mediated by the potentiation of GABA-A receptors [43]. Although the effect of these substances increasing the beta power measured with EEG could not be related to its anti-tremorogenic effects, we observed a significant relationship between an increase in the ratio of beta to tremor-related cortical activity and a reduction in contralateral postural tremor. Indeed, we observed this dependency for each patient individually (Fig. 4). We acknowledge that this finding may be an epiphenomenon or a consequence, albeit not necessarily direct, of the biochemical effect of alprazolam on the brain. Nevertheless, considering that during sustained motor contraction the activity within cortical motor areas and their target muscles is synchronised in the beta-range [22], [23], [44]–[46], we hypothesise that the increased physiological beta activity in the primary motor cortex may be interfering, at least in part, with the coupling of pathological oscillatory networks involved in the generation of tremor in ET.

Since EEG technology has limited spatial resolution, it cannot be stated from our data that the cortical changes observed at the tremor frequency after alprazolam intake are exclusively due to changes in the tor cortex. Indeed, previous studies on cortico-muscular coherence in ET have pointed to a combined effect of the afferent and efferent tracts [7]. It has also been shown that existing techniques to detect the actual direction of information flow are not always precise enough [47]. The main purpose of the present study is to characterize the interaction between the central (cortical) rhythms and the peripheral tremor. Indeed, we also observed a significant correlation between the tremor reduction and the increase in cortical beta activity by the effects of alprazolam alone (results no included here). In that case, the significance of the correlation was slightly smaller than the one obtained using the ratio between the beta and the tremor-related cortical activities but also suggested that there are changes in cortical beta activity when tremor is reduced.

There is increasing evidence suggesting that Purkinje cells (PC) and connected neuronal populations are centrally involved in the generation of tremor in ET [42], [48]–[51]. Regardless of whether ET is a neurodegenerative disease affecting PC [51], or the result of pacemaking neurons in the inferior olive [48]–[50], these structures are controlled by GABAergic connections [52]. Recently, Paris-Robidas et al. reported a post-mortem decrease in GABA receptors in the dentate nucleus of the cerebellum from individuals with ET (compared with controls or individuals with Parkinson’s disease) that could result in disinhibition of cerebellar pacemaker output activity [53]. Therefore, we are aware that the GABAergic effect of alprazolam would not be restricted to the sensorimotor cortex but could be spread through cerebello-thalamic pathways. Indeed, localised microinjections of the GABA-A agonist muscimol into the ventral intermediate nucleus (in areas with electrophysiologically identified tremor-synchronous cells) of ET patients undergoing stereotaxy were effective at reducing tremor [54].

Results presented here could be of pathophysiological interest, providing biomarkers for alprazolam induced cortical activity modulation in vivo. We also consider that the interplay between cortical activity in the beta band and pathological tremor-related cortical activity could be an easily measurable marker of efficiency, providing new tools to assess the effect of novel therapeutic strategies that enhance GABAergic inhibition with other mechanisms instead of benzodiazepines.

Finally, the authors acknowledge several limitations of the present study but consider that their impact on the conclusions is minor. First, we recruited a small group of patients and thus our results might not be generalisable to a broader population of ET cases. However, the homogeneity of the results obtained from all the patients separately (Fig. 5 shows that the identified positive tendency is present in all cases) reinforces the hypothesis proposed in this study. Second, we did not use a placebo group. However, our data do not indicate that any of the patients experienced placebo effects given that the observed reduction in tremor severity 4 min after alprazolam intake was negligible compared to subsequent runs (Fig. 1). We consider that the results were not influenced by expectancy bias because the patients had never received alprazolam as treatment before and were unfamiliar with the therapeutic effects of the drug to alleviate tremors.

In conclusion, we have shown that alprazolam attenuates tremors in ET and increases the ratio between the beta and tremor-related cortical activity as well as decreases the strength of cortico-muscular coupling at the tremor frequency. We hypothesise that the increase in cortical beta activity due to the effects of alprazolam acts as a blocking mechanism of activity in pathological neural networks, which leads to a reduction of tremors in ET patients. This is the first study to describe the neurophysiological changes occurring in ET patients after benzodiazepine administration, and it is expected that future experiments will identify other drugs that reduce tremors in ET and that will enhance our understanding of the pathophysiology of this disease and its response to different treatments.
